# Up in Flames: The Safety of Electrocautery Trephination of Subungual Hematomas with Acrylic Nails

**DOI:** 10.5811/westjem.2021.10.53567

**Published:** 2022-02-23

**Authors:** Claude Blereau, Steven Radloff, Justin Grisham

**Affiliations:** * Madigan Army Medical Center, Department of Emergency Medicine, Joint Base Lewis-McChord, Washington; † Evans Army Community Hospital, Department of Emergency Medicine, Fort Carson, Colorado

## Abstract

**Introduction:**

Subungual hematomas are fingertip injuries, generally secondary to blunt trauma, that cause pain due to an accumulation of blood under the fingernail. It is generally considered standard of practice to relieve this accumulation by means of trephination with a hollow tip needle, a heated paper clip, or electrocautery. It has been assumed that due to the flammable properties of acrylic, trephination via electrocautery has the potential to ignite acrylic nails and cause burns and other potentially serious injury, making electrocautery contraindicated in patients with acrylic nails. Our thorough literature review failed to support or refute this assumption; so in the interest of ensuring that this practice is evidence-based, we sought to explore this topic.

**Methods:**

In this study we used electrocautery trephination on acrylic nail products attached to simulated digits and recorded the presence and frequency of ignition events. We hypothesized that ignition would occur with sufficient frequency to support continuing the practice of avoiding electrocautery trephination in subungual hematomas with overlying acrylic nails.

**Results:**

In our study, we exposed 200 acrylic nails to trephination with electrocautery, and 83 nails ignited (41.5%).

**Conclusion:**

While other variables exist, these findings do support the current practice pattern of avoiding trephination with electrocautery in those patients with acrylic nails overlying subungual hematomas.

## INTRODUCTION

A subungual hematoma is an accumulation of blood under the fingernail that develops when damage occurs to the richly vascular nail bed, generally as the result of a blunt trauma or crush injury to that nail bed.^1^,^2^ These injuries present to the emergency department (ED) frequently^3^,^4^; they classically present with pain and a dark discoloration under the nail following a minor crush injury to the distal finger. The pain is reduced by relieving the pressure built up between the nail and the distal phalanx.^2^ Until relatively recently, the recommended process of evaluation and treatment for these injuries frequently included removal of the nail to evaluate the underlying nail bed for laceration.^5^ Recent studies have shown trephination of the nail to be as efficacious and without additional risk, and trephination with a hollow tip needle, a heated paper clip, or electrocautery has become the standard of practice.^3^,^5^

The presence of acrylic nails complicates treatment and evaluation of distal finger injuries in several ways. First, visualization of the nail bed is obviously limited. There has also been speculation that the presence of infectious agents in acrylic nails has the potential to cause harm.^6^ Additionally, it has been assumed that due to the flammable properties of acrylic,^7^ trephination via electrocautery has the potential to ignite acrylic nails and cause burns as well as other serious injury.^2^ In this study we sought to explore this assumption, as a thorough literature review found no prior studies to either support or refute it. We exposed acrylic nail products with simulated digits to a common form of electrocautery pen used in the ED, the Bovie electrocautery pen (Symmetry Surgical Inc., Antioch, TN), and recorded the presence and frequency of adverse events. We hypothesized that ignition would occur with sufficient frequency to make the use of electrocautery for trephination of subungual hematomas with overlying acrylic nails ill advised.

## METHODS

We conducted a non-randomized experimental trial. The desired outcome was to determine the likelihood of combustion involved with the use of electrocautery for trephination of acrylic nails. No human or animal subjects were used, and no patient data was collected; this study received approval from the Madigan Institutional Review Board. We created faux fingers with acrylic fingernails using consumer-available hot dogs and a “do-it-yourself” acrylic nail kit: KISS French Acrylic Sculpture Kit (KISS Products Inc, Port Washington, NY). The manufacturer’s instructions were followed,^8^ other than that the acrylic nails were applied to the faux fingers rather than human fingernails. Using the kit’s provided glue, we secured the plastic fingernails to the simulated digits made from pieces of a hot dog cut longitudinally and transversely to mimic fingertips. After allowing the glue adequate time to dry, per the manufacturer’s instructions, we mixed the acrylic liquid and polymer powder and applied it to each nail with a focus on distributing the acrylic mix evenly. A total of 200 fingernails were assembled for this study.

The fabricated fingernails were then allowed to harden for 24 hours prior to the trephination trials with electrocautery pens. We used the high temperature, battery-operated Bovie electrocautery pen (Symmetry Surgical Inc., Antioch, TN) throughout the trial. In total, seven electrocautery pens were used during the trephination of the 200 acrylic nails. Each trial consisted of the electrocautery pen being turned on and allowed to reach maximum temperature, which was considered to be achieved when the electrocautery tip reached a white-hot glow. Trephination of the acrylic nail was performed at a perpendicular angle until the tip of the electrocautery pen completely penetrated through the plastic fingernail (Figure). The results were recorded on a data sheet as “Ignition” or “No Ignition.” Each electrocautery pen was used until it failed to reach maximum temperature as defined above. Once all fingernails were used, we compiled and processed the data to determine the percentage of fingernails that ignited.

## RESULTS

Of the total 200 acrylic nails exposed to electrocautery trephination in this study (n = 200), 83 ignited (Table). This represents 41.5% combustion when electrocautery was used. Each of seven Bovie pens was used for multiple trephinations to reduce waste and cost. Of note, the percentage of acrylic nails that ignited due to trephination varied widely from pen to pen, with the highest percentage being 76.2% and the lowest 13.0%. Early in our study we did not record which pens were used. For the first 84 trephinations, we used three pens, none of which were used again thereafter. We did not record which of those three pens was used to demonstrate either ignition or no ignition. For the remaining 116 trephinations, four pens were used. We recorded the incidence of ignition for each of the four pens (Table).

## DISCUSSION

We used disposable electocautery pens multiple times, which revealed a wide range of variation between them in the number of nails ignited. This was a relatively unexpected finding, and not the primary outcome of our study; thus, early in the data-gathering phase of our study, we did not record the particular pen used. As we began to notice that some electrocautery devices had a notably higher rate of ignition than others, we began to record which of four Bovie pens was used in each of the trephinations. The variation in ignition rates per pen was likely due to variation in the temperature achieved by the pens both inherently and as the single-use pens continued to be used. Nevertheless, the percentage of acrylic nails that were ignited during trephination was clinically significant and provides evidence to support the current practice of avoiding electrocautery for trephination as treatment for subungual hematomas in the presence of acrylic nails.

## LIMITATIONS

This was a relatively small study conducted using only one brand of do-it-yourself acrylic nail kit and store-bought hot dogs to represent human digits. There are minor differences in the application process and the chemicals used in other brands, as well as in professionally applied acrylic nails. The rate of ignition may vary as a result of these differences. Additionally, there were significant differences in the percentage of ignitions caused by different electrocautery pens, although we used all the same type and brand. This implies the possibility that there may be a temperature range at which electrocautery is both safer for use with acrylic and capable of trephination. While further studies could be conducted to determine this, the presence of other treatment options, and the relative paucity of patients having both subungual hematomas and acrylic nails, may limit the utility of further investigation in the future.

## CONCLUSION

Subungual hematomas are relatively common blunt or crush injuries to the fingertip that are presented to the ED. Treatment by trephination of the nail has become the standard of practice, and the use of electro-cautery as a means of trephination is common. Use of electrocautery for trephination of acrylic nails has long been thought to be contraindicated due to the known propensity for acrylic to ignite; however, there have been no previous studies to validate this assumption. Our study demonstrated a rate of ignition of 41.5% when electrocautery pens were used in the trephination of 200 acrylic nails on simulated human digits. While the sample size is small, the high incidence of ignition validates the current practice pattern of avoiding electrocautery for trephination of the nail bed as treatment for subungual hematomas when acrylic nails are present.

## Figures and Tables

**Figure:**
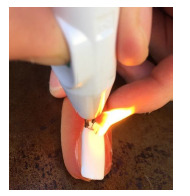
Acrylic nail igniting on simulated fingertip after trephination with an electrocautery pen.

**Table:** Incidence of acrylic nail ignition when using electrocautery pen.

Electrocautery pen #	Unknown		1		2		3		4		Total	
					
	I	NI	I	NI	I	NI	I	NI	I	NI	I	NI
	27	57	7	14	3	20	34	17	16	5	83	117
Total	84		21		23		51		21		200	
Percentage	32.1	67.9	33.3	66.7	13.0	87.0	66.7	33.3	76.2	23.8	41.5	58.5

*I,* ignition; *NI,* no ignition.

## References

[1] Salter S, Ciocon D, Gowrishankar T (2006). Controlled nail trephination for subungual hematoma. Am J Emerg Med.

[2] Pirzada A, Waseem M (2004). Subungual hematoma. Pediatr Rev.

[3] Seaberg D, Angelos W, Paris P (1991). Treatment of subungual hematomas with nail trephination: a prospective study. Am J Emerg Med.

[4] Fetter-Zarzeka A, Joseph M (2002). Hand and fingertip injuries in children. Pediatr Emerg Care.

[5] Dean B, Becker G, Little C (2012). The management of the acute traumatic subungual haematoma: a systematic review. Hand Surgery.

[6] Gil J, DeFroda S, Reid D (2016). Closed traumatic finger tip injuries in patients with artificial nails: removal of UV gel and acrylic nails. Am J Emerg Med.

[7] Vanover W, Woods J, Allin S (1999). Synthetic fingernails as a fire hazard in the chemistry laboratory. J Chem Educ.

[8] Search results for: ‘nails kiss french’. Kissusa.com.

